# Effect of desflurane-remifentanil vs. Propofol-remifentanil anesthesia on arterial oxygenation during one-lung ventilation for thoracoscopic surgery: a prospective randomized trial

**DOI:** 10.1186/s12871-017-0302-x

**Published:** 2017-01-18

**Authors:** Youn Joung Cho, Tae Kyong Kim, Deok Man Hong, Jeong-Hwa Seo, Jae-Hyon Bahk, Yunseok Jeon

**Affiliations:** Department of Anesthesiology and Pain Medicine, Seoul National University Hospital, 101 Daehak-ro, Jongno-gu, Seoul, 03068 South Korea

**Keywords:** Desflurane, Propofol, One-lung ventilation, Oxygenation

## Abstract

**Background:**

One-lung ventilation during thoracic surgery frequently disturbs normal systemic oxygenation. However, the effect of anesthetics on arterial oxygenation during one-lung ventilation has not been well established in human study. In this clinical trial, we investigated whether a difference between desflurane-remifentanil and propofol-remifentanil anesthesia can be observed with regard to oxygenation during one-lung ventilation for thoracoscopic surgery.

**Methods:**

Adult patients with lung cancer, scheduled for video-assisted thoracoscopic lobectomy without preoperative oxygen support, were screened and randomized to receive desflurane or propofol, with remifentanil continuous infusion in both groups. Mechanical ventilation was performed with tidal volume of 8 ml/kg and F_I_O_2_ 0.5 during two-lung ventilation, and 6 ml/kg and 1.0 during one-lung ventilation, both with positive end-expiratory pressure of 5 cmH_2_O. Arterial blood gas analysis was performed preoperatively, during two-lung ventilation, and after 15, 30, 45, and 60 min of one-lung ventilation. The primary endpoint was PaO_2_ at 30 min after initiating one-lung ventilation. Statistical analyses included the independent *t*-test for the primary endpoint and a mixed model with a post-hoc analysis to evaluate the serial changes in values.

**Results:**

Patients were recruited between July 9 and December 2, 2014. In total, 103 patients were analyzed (*n* = 52 in desflurane group and *n* = 51 in propofol group). The primary endpoint, PaO_2_ at 30 min of one-lung ventilation was lower in the desflurane group than the propofol group (170 ± 72 vs. 202 ± 82 mmHg; *p* = 0.039). Serial changes in PaO_2_ during one-lung ventilation showed lower levels during desflurane anesthesia compared with propofol anesthesia (mean difference, 45 mmHg; 95% confidence interval, 16–75 mmHg; *p* = 0.003).

**Conclusions:**

In conclusion, desflurane-remifentanil anesthesia resulted in decreased arterial oxygenation compared with that of propofol-remifentanil anesthesia during one-lung ventilation for thoracoscopic surgery in patients with lung cancer.

**Trial registration:**

ClinicalTrials.gov identifier: NCT02191371, registered on July 7, 2014

## Background

One-lung ventilation (OLV) during thoracic surgery can rapidly result in impaired systemic oxygenation. The grade of hypoxemia during OLV is mainly influenced by an increase in shunt and dead space. Hypoxic pulmonary vasoconstriction (HPV) is a physiological protective mechanism that diverts pulmonary perfusion from the non-ventilated to the ventilated area of the lung, thereby decreasing the shunt of unsaturated blood and ameliorating the degree of hypoxemia. This physiologic modulation is influenced by numerous factors such as various drugs, temperature, acid–base status, airway pressure, patient position, and cardiac output [1]. In numerous animal studies and in studies of isolated lungs, HPV has been shown to be modulated by volatile anesthetics [1–5]. However, human studies have yielded inconsistent results regarding the effects of different anesthetics on systemic oxygenation during OLV [6–10]. Moreover, the effects of desflurane-remifentanil balanced anesthesia have not been evaluated in a clinical study.

Most halogenated inhaled anesthetics are characterized by its dose-dependent systemic vasodilatory effect [11–14]. We hypothesized that the vasodilatory effect of desflurane may affect any protective role of HPV, thus impair oxygenation during OLV, even when it is used as a balanced anesthesia, compared with total intravenous anesthesia (TIVA). To evaluate this hypothesis, we compared the effects of two commonly used modern anesthetics, desflurane and propofol, with concomitant remifentanil continuous infusion in both arms, on systemic oxygenation during OLV for video-assisted thoracoscopic surgery.

## Methods

The Institutional Review Board of Seoul National University Hospital approved this study (reference # 1406-052-587; approval date, July 7, 2014). All patients provided written informed consent, and the study was performed according to Good Clinical Practice guidelines and the principles of the Declaration of Helsinki (ClinicalTrials.gov identifier: NCT02191371; principal investigator, Yunseok Jeon; registered on July 7, 2014).

### Patient inclusion and randomization

Patients with lung cancer scheduled for elective video-assisted thoracoscopic lobectomy requiring OLV at Seoul National University Hospital were screened for eligibility. Exclusion criteria were age < 20 years, ASA class > III, BMI > 30 kg/m^2^, symptomatic or severe obstructive or restrictive lung disease, requirement for preoperative oxygen supply, preoperative intubated state or under mechanical ventilatory support, baseline arterial partial pressure of oxygen (PaO_2_) < 70 mmHg, symptomatic coronary or peripheral arterial disease, renal failure, preoperative continuous infusion of inotropes or vasopressor, refuse to participate in the study, and pregnancy. The interpretation of the pulmonary function test results was based on the American Thoracic Society/European Respiratory Society guidelines [15]. Obstructive disease was defined when forced expiratory volume in 1 s (FEV_1_)/forced vital capacity (FVC) was < 0.7, and severity was determined by % predicted FEV_1_ value; severe disease was diagnosed when FEV_1_ < 50% predicted value.

After receiving informed consent, eligible patients were randomized to receive either inhalational anesthesia with desflurane (Suprane®, Baxter Healthcare, Deerfield, IL) or intravenous anesthesia with propofol (Fresofol®2 MCT 2%, Fresenius Kabi, Graz, Austria) as a main anesthetic, in the morning of the day of surgery. Block randomization (blocks of 4 or 6) was performed before assigning the groups using a computer-generated randomization program operated by a clinician not involved in the study. Baseline pulmonary function, blood pressure and heart rate were recorded one day before surgery.

### Study protocol

Standardized pre-surgical and surgical procedures were described previously [16]. Three-lead electrocardiography, pulse oximetry (SpO_2_), and non-invasive blood pressure monitoring were performed in all patients. Without premedication, general anesthesia was induced with target-controlled infusion of propofol (starting with a target effect site concentration, Ce of 4 μg/ml) in the propofol group. In the desflurane group, we avoided the use of propofol even during the induction period. Therefore, we used etomidate (Etomidate® Lipuro, 0.25 mg/kg; B. Braun, Melsungen, Germany) to induce anesthesia to exclude any potential effects of propofol in the desflurane group. Anesthesia was maintained with either desflurane (5–7 vol%) or propofol (Ce of 3–4 μg/ml), and all patients were continuously infused with remifentanil (Ce of 1.5–3.5 ng/ml, Ultiva™; GlaxoSmithKline, San Polo di-Torrile, Italy) during anesthesia induction and throughout the surgery. Administration of anesthetics (desflurane or propofol) was titrated to maintain appropriate anesthetic depth [bispectral index (BIS), 30–50] using electroencephalogram-based hypnotic monitor (BIS VISTA™ monitor, Aspect Medical Systems, Norwood, MA). In both groups, infusion of remifentanil was adjusted according to hemodynamic changes. A commercial infusion pump (Orchestra®; Fresenius Vial, Brezins, France) was used for target-controlled infusion of propofol and remifentanil according to the patients’ demographic data (sex, age, height and weight). The techniques for anesthesia maintenance in our study were based on those described in previous studies and textbooks [17–20]. Infusion of propofol was monitored with the Marsh pharmacokinetic model and remifentanil with the Minto model.

After establishing neuromuscular blockade with administration of rocuronium (0.6–0.8 mg/kg), the patient’s trachea was intubated with a left or right double-lumen endotracheal tube (Mallinckrodt™ endobronchial tube, size 32–39 Fr; Covidien, Mansfield, MA). The size of the tube was chosen based on the patient’s height and mainstem bronchial diameter, and the correct position of the tube was confirmed with a flexible fiber-optic bronchoscope. The lungs were mechanically ventilated with volume-controlled ventilation with a tidal volume of 8 ml/kg (predicted body weight, PBW) during two-lung ventilation (TLV) and 6 ml/kg (PBW) during OLV. The inspired oxygen fraction (F_I_O_2_) was set to 0.5 during TLV and 1.0 during OLV. After the patients were positioned in the lateral decubitus position, the endotracheal tube position was rechecked using a fiber-optic bronchoscope before initiation of OLV. Respiratory rate was started at 12/min and adjusted to maintain a carbon dioxide arterial partial pressure of between 35 and 45 mmHg. Positive end-expiratory pressure was maintained at 5 cmH_2_O.

The right or left radial artery was cannulated with a 20-G Angiocath™ (Becton Dickinson Medical Ltd., Tuas, Singapore) and connected to a FloTrac™ transducer with an EV1000™ monitor (Edwards Lifesciences LLC, Irvine, CA). Arterial blood pressure and cardiac index by pulse-contour analysis were monitored continuously, and arterial blood sampling for gas analysis was conducted through the arterial cannula. A central venous catheter (Multi-Med; Edwards Lifesciences) was placed at either the right or left internal jugular vein under ultrasonographic guidance. Crystalloid or colloid was administered to maintain adequate hemodynamics by the attending anesthesiologist. A paravertebral and/or intercostal nerve block was performed at the surgeon’s discretion before the surgery was completed. Intravenous patient-controlled analgesia with morphine and fentanyl was provided to all patients postoperatively according to their demographic data and the extent of the surgery.

### Data collection

Hemodynamic recording and arterial blood gas analysis were conducted at the following time points: (1) preoperatively, (2) TLV before OLV, (3) 15, (4) 30, (5) 45, and (6) 60 min after initiation of OLV. Preoperative data were obtained on room air one day prior to surgery. To minimize the effect of the patient’s position, data during TLV were obtained in the lateral decubitus position. Arterial blood gas analysis was performed using a GEM® Premier 3000 (Model 5700; Instrumentation Laboratory, Lexington, MA) within 3 min after obtaining the blood sample. We restricted recruitment maneuvers to the ventilated lung during OLV, unless there was unacceptable desaturation (defined as SpO_2_ < 90% or PaO_2_ < 70 mmHg at F_I_O_2_ 1.0) or difficulty in maintaining OLV without additional manipulation. If additional manipulation was required, subsequent data were excluded from the analysis to avoid any confounding effects.

### Primary endpoint and power analysis

The primary endpoint was PaO_2_ at 30 min of OLV. In a previous study that investigated oxygenation during OLV in patients receiving propofol, mean ± SD PaO_2_ at 30 min of OLV was 28 ± 9 kPa (209 ± 68 mmHg) [21]. We calculated that 52 patients would be required per group to detect a PaO_2_ difference of 20% after 30 min of OLV between the groups, as compared using an independent *t*-test, including a 20% dropout rate, using a two-sided design at a significance level of 5% and 80% power (G*Power ver. 3.1.9.2; Franz Faul, Universitat Kiel, Germany).

### Statistical analyses

Data are presented as the means ± SD, numbers (%), or median (95% confidence interval). Normality of the data was checked using the Kolmogorov–Smirnov and Shapiro–Wilk tests. Residuals vs. fitted values plots were used for repeated-measured variables to check that the error terms (residuals) had a mean of zero and constant variance. The plots showed a pattern consistent with equal variances. The normality assumption for the model residuals was checked with histograms and normal quantile-quantile (Q-Q) plots of the residuals, and the data were determined to be normally distributed. Comparisons were conducted using the independent *t*-test or Mann–Whitney *U*-test for continuous variables, and Pearson’s chi-squared test or Fisher’s exact test for non-continuous variables. Sequential changes of variables were analysed using linear mixed models with Bonferroni’s correction. In the mixed model, the treatment group, time, and the interaction between group and time were regarded as fixed effects, and subject was regarded as a random effect. Analysis was performed using SPSS software (ver. 21.0.0.0 for Windows; IBM, Armonk, NY). In all analysis, *p* <0.05 was taken to indicate statistical significance.

## Results

**Fig. 1:**
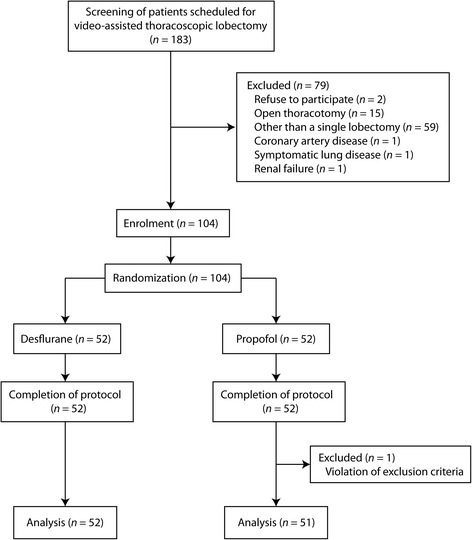
CONSORT diagram of study flow

Patient recruitment and data collection was performed between July and December 2014. Among the 183 patients with lung cancer screened for eligibility, 104 patients were randomized to either desflurane (*n* = 52) or propofol group (*n* = 52; Fig. 1). After completion of protocol, 103 patients (52 in the desflurane and 51 in the propofol group) were analyzed. One patient in the propofol group was excluded from the primary analysis, as her baseline PaO_2_ was lower than the exclusion criteria (70 mmHg). The characteristics of the patients included in this study were well balanced between the groups (Table 1). There was no difference in underlying diseases and current medications between groups. We included patients scheduled for a lobectomy at enrolment, but several patients required procedures other than pre-planned lobectomy during surgery (Table 1). The distribution of operation types was comparable between the groups (*p* = 0.428). The right lung was ventilated during OLV in 32/52 and 34/51 patients in the desflurane and propofol groups, respectively (*p* = 0.682).

**Table 1:** Characteristics of patients receiving desflurane-remifentanil or propofol-remifentanil during one-lung ventilation for thoracoscopic surgery

	Desflurane group (*n* = 52)	Propofol group (*n* = 51)	*p* value
Female	18 (35%)	23 (45%)	0.277
Age (year)	63 ± 8	62 ± 10	0.841
Body mass index (kg/m^2^)	24.0 ± 2.8	22.9 ± 2.8	0.050
ASA class			0.914
I	24 (46%)	23 (45%)	
II	28 (54%)	28 (55%)	
Smoking history			0.257
Never smoker	28 (54%)	28 (55%)	
Current smoker	11 (21%)	16 (31%)	
Former smoker	13 (25%)	7 (14%)	
Preoperative hemoglobin (g/dl)	13.2 ± 1.3	13.2 ± 1.3	0.820
Preoperative FEV_1_ (% predicted)	100.6 ± 16.3	103.8 ± 19.0	0.356
Preoperative FVC (% predicted)	98.0 ± 12.6	100.2 ± 13.7	0.391
Preoperative PaO_2_ (mmHg)	92 ± 14	93 ± 11	0.798
Preoperative medications			
Aspirin	7 (14%)	8 (16%)	0.749
COX inhibitor other than aspirin	2 (4%)	4 (8%)	0.437
Calcium antagonist	12 (23%)	11 (22%)	0.854
ACE inhibitor	0 (0%)	1 (2%)	0.495
ARB	10 (20%)	13 (26%)	0.446
Beta blocker	2 (4%)	3 (6%)	0.678
Diuretics	2 (4%)	6 (12%)	0.160
OHA	7 (14%)	9 (18%)	0.558
Clopidogrel	0 (0%)	2 (4%)	0.243
Statin	8 (15%)	14 (28%)	0.135
Steroid	0 (0%)	2 (4%)	0.243
Prostacyclin	0 (0%)	1 (2%)	0.495
Anesthesia time (min)	222 ± 47	211 ± 46	0.243
Infused crystalloid (ml)	940 ± 505	784 ± 378	0.079
Infused colloid (ml)	117 ± 192	99 ± 167	0.600
Urinary output (ml)	192 ± 159	201 ± 152	0.769
Estimated blood loss (ml)	160 ± 89	143 ± 75	0.299
Extent of operation			0.428
Lobectomy	41 (79%)	44 (86%)	
Segmentectomy	7 (13%)	3 (6%)	
Wedge resection	4 (8%)	4 (8%)	
Mainly resected lobe			0.747
Right upper lobe	16 (31%)	15 (29%)	
Right middle lobe	3 (6%)	7 (14%)	
Right lower lobe	13 (25%)	12 (23%)	
Left upper lobe	10 (19%)	8 (16%)	
Left lower lobe	10 (19%)	9 (18%)	

*FEV*_*1*_ forced expiratory volume in 1 s, *FVC* forced vital capacity, *PaO*_*2*_ arterial partial pressure of oxygen, *COX* cyclooxygenase, *ACE* angiotensin converting enzyme, *ARB* angiotensin receptor blocker, *OHA* oral hypoglycemic agent. Preoperative FEV_1_ and FVC are expressed as percentages of the predicted value. Values are numbers (%) or means (SD)

**Fig. 2:**
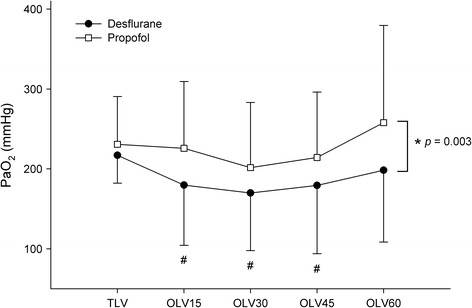
Arterial oxygenation during desflurane-remifentanil vs. propofol-remifentanil anesthesia for one-lung ventilation. Data points are mean; error bars are SD. PaO_2_ during OLV was lower in the desflurane group than the propofol group. During desflurane anesthesia, PaO_2_ was significantly lower at 15, 30, and 45 min of OLV compared to the TLV state. PaO_2_, arterial partial pressure of oxygen; TLV, two-lung ventilation; OLV, one-lung ventilation; OLV15, 15 min of OLV; OLV30, 30 min of OLV; OLV45, 45 min of OLV; OLV60, 60 min of OLV. * *p* <0.01 between the two groups during OLV period (linear mixed model). # Adjusted *p* <0.01 when compared to the TLV state in the desflurane group (linear mixed model with Bonferroni’s correction)

Intraoperative blood gas and anesthetic variables at each time point in each group are shown in Table 2. Preoperative and pre-OLV (during TLV before initiation of OLV) PaO_2_ were comparable between the groups (92 ± 14 vs. 93 ± 11 mmHg and 217 ± 35 vs. 231 ± 60 mmHg in the desflurane vs. propofol group, respectively). Three pre-OLV PaO_2_ data (2 in the propofol group and 1 in the desflurane group) were excluded from analysis, as F_I_O_2_ (0.5) was not maintained during the period. The primary endpoint, PaO_2_ at 30 min of OLV was lower in the desflurane group than the propofol group (170 ± 72 vs. 202 ± 82 mmHg in desflurane vs. propofol group, respectively; *p* = 0.039). For other time points during OLV, PaO_2_ was lower in the desflurane group compared to the propofol group [180 ± 75 vs. 226 ± 84 mmHg (*p* = 0.004), 179 ± 85 vs. 214 ± 82 mmHg (*p* = 0.043), and 198 ± 90 vs. 258 ± 122 mmHg (*p* = 0.009) in desflurane vs. propofol group at 15, 45, and 60 min of OLV, respectively; Fig. 2]. The lowest PaO_2_ during the OLV period was lower in the desflurane group than the propofol group (133 ± 50 vs. 162 ± 63 mmHg, respectively; *p* = 0.010). The serial changes in PaO_2_ during 60 min of OLV revealed a lower PaO_2_ in the desflurane group compared to that in the propofol group (mean difference, 45 [95% CI, 16–75] mmHg; *p* = 0.003). The interaction between group and time was not significant during OLV (*p* = 0.098). Compared to the pre-OLV state, PaO_2_ at 15, 30, and 45 min of OLV were significantly lower in the desflurane group (217 ± 35 vs. 180 ± 75, 170 ± 72, and 179 ± 85 mmHg; adjusted *p* = 0.003, *p* < 0.001, and *p* = 0.002, respectively), but not in the propofol group at any time point (Fig. 2).

**Table 2:** Intraoperative blood gas and anesthetic variables in patients receiving desflurane-remifentanil or propofol-remifentanil during one-lung ventilation

	TLV	15 min of OLV	30 min of OLV	45 min of OLV	60 min of OLV
Desflurane group (*n* = 52)					
End-tidal desflurane (vol%)	5.4 ± 1.0	6.2 ± 0.8	6.2 ± 0.8	6.2 ± 0.8	6.2 ± 0.8
Minimum alveolar concentration	1.0 ± 0.2	1.1 ± 0.2	1.1 ± 0.2	1.1 ± 0.1	1.1 ± 0.2
Ce, remifentanil (ng/ml)	2.3 ± 1.1	2.4 ± 0.9*	2.1 ± 1.1*	1.9 ± 1.0*	1.8 ± 1.0*
MAP (mmHg)	84 ± 13	85 ± 15	78 ± 12*	75 ± 12*	78 ± 9*
HR (beats/min)	68 ± 13	67 ± 11	70 ± 11	68 ± 10	69 ± 12
BT (°C)	36.1 ± 0.4	36.1 ± 0.4	36.0 ± 0.4	35.9 ± 0.4	35.8 ± 0.5
CVP (mmHg)	7 ± 3	8 ± 3	7 ± 3	7 ± 3	7 ± 3
CI (l/min/m^2^)	2.7 ± 0.6	2.6 ± 0.6	2.9 ± 0.7	2.9 ± 0.7	2.9 ± 0.7
P_peak_ (cmH_2_O)	19 ± 2	22 ± 3	23 ± 4	23 ± 3	23 ± 3
PaCO_2_ (mmHg)	41 ± 4	45 ± 5	45 ± 4	45 ± 4	45 ± 5
Arterial blood pH	7.4 ± 0.0	7.4 ± 0.0	7.4 ± 0.0	7.4 ± 0.0	7.4 ± 0.0
Base excess (mmol/l)	2.1 ± 2.2	1.9 ± 2.1	1.5 ± 2.2	1.6 ± 2.0	1.5 ± 2.1
Propofol group (*n* = 51)					
Ce, propofol (μg/ml)	3.6 ± 0.6	3.6 ± 0.6	3.5 ± 0.5	3.5 ± 0.5	3.6 ± 0.5
Ce, remifentanil (ng/ml)	2.5 ± 1.2	3.0 ± 1.3*	3.1 ± 1.3*	3.2 ± 1.1*	3.3 ± 1.1*
MAP (mmHg)	82 ± 13	85 ± 13	84 ± 10*	81 ± 10*	83 ± 10*
HR (beats/min)	64 ± 11	65 ± 12	68 ± 12	65 ± 11	65 ± 11
BT (°C)	36.0 ± 0.4	35.9 ± 0.4	35.9 ± 0.4	35.8 ± 0.4	35.8 ± 0.4
CVP (mmHg)	6 ± 3	7 ± 3	7 ± 3	7 ± 4	7 ± 3
CI (l/min/m)	2.6 ± 0.7	2.7 ± 0.7	2.8 ± 0.9	2.8 ± 0.8	2.9 ± 0.8
P_peak_ (cmH_2_O)	19 ± 2	21 ± 3	23 ± 3	23 ± 3	23 ± 3
PaCO_2_ (mmHg)	42 ± 5	44 ± 5	45 ± 5	44 ± 5	44 ± 5
Arterial blood pH	7.4 ± 0.0	7.4 ± 0.0	7.4 ± 0.0	7.4 ± 0.0	7.4 ± 0.0
Base excess (mmol/l)	2.5 ± 1.9	2.3 ± 2.0	2.2 ± 2.0	2.0 ± 2.0	2.0 ± 1.9

*TLV* two-lung ventilation, *OLV* one-lung ventilation, *Ce* effect-site concentration, *MAP* mean arterial pressure, *HR* heart rate, *BT* body temperature, *CVP* central venous pressure, *CI* cardiac index, *P*_*peak*_ peak airway pressure, *PaCO*_*2*_ arterial partial pressure of carbon dioxide. Values are means (SD)

* *p* <0.05 between the groups

After 30 min of OLV, mean arterial pressure (MAP) was lower in the desflurane group, and accordingly, lesser remifentanil was used in the desflurane group than propofol group (Table 2). The total amounts of remifentanil administered during surgery were 911 ± 337 μg in the desflurane group and 1407 ± 638 μg in the propofol group, respectively (*p* <0.001). BIS was maintained within the target range (30–50) throughout the surgery in both groups.

Two patients in the desflurane group received continuous infusion of a vasoactive drug (noradrenaline and phenylephrine, respectively) to maintain adequate hemodynamics. None of the patients received transfusion peri-operatively. Three cases in the desflurane group (*p* = 0.243) required lung recruitment strategies during OLV to maintain an adequate oxygenation profile, and therefore subsequent data were excluded from comparison of PaO_2_ in these patients after the recruitment maneuvers. The rescue maneuvers were performed after 30 min of OLV in all three cases. After alveolar recruitment, hypoxemia was improved and no changes in the anesthetic protocol were required.

## Discussion

In this study, oxygenation under desflurane-remifentanil balanced anesthesia was lower than that under propofol-remifentanil TIVA during OLV for video-assisted thoracoscopic surgery. This is the first reported clinical trial in which desflurane was shown to impair oxygenation during OLV compared to TIVA in lung cancer patients undergoing thoracoscopic lung surgery.

Recently, many surgical procedures have been performed using less-invasive approaches. Large percentages of thoracic operations for lung resection are performed through video-assisted thoracoscopic surgery. Thus, the durations of both the operation and postoperative recovery period have been shortened. Both desflurane and propofol are relatively new anesthetics with short-acting properties characterized by rapid onset and offset due to their low blood gas partition coefficient and low context-sensitive half time, respectively. Therefore, they are highly attractive and offer fast-track anesthesia and earlier recovery for postoperative rehabilitation in minimal invasive thoracic surgery. However, the roles of these modern anesthetics in management of oxygenation during OLV remain unclear in humans.

In animal studies, inhalational anesthetics are known to inhibit the protective role of HPV during alveolar hypoxia combined with OLV both in vitro and in vivo [2]. Halothane, enflurane, isoflurane, sevoflurane, and desflurane depressed HPV in a dose-dependent manner in isolated rat or rabbit lung [4, 22, 23]. Isoflurane or desflurane impaired oxygenation during OLV, while intravenous anesthetics, such as propofol or pentobarbital, had no detrimental impact on HPV in animal studies [3, 5, 24, 25]. However, in clinical investigations, results regarding the effects of anesthetics on oxygenation and HPV during OLV have been inconsistent [6–10, 26, 27]. Propofol improved oxygenation and shunt fraction during OLV compared to sevoflurane anesthesia in patients undergoing esophagectomy [6] or thoracotomy pulmonary lobectomy [27]. In other studies, propofol anesthesia did not differ in changes in shunt fraction or oxygenation during OLV for thoracic surgery in comparison with sevoflurane or isoflurane [7–10]. Moreover, arterial oxygenation was not different between propofol-alfentanil vs. isoflurane anesthesia during OLV in patients undergoing thoracoscopic pulmonary surgery or esophageal surgery [26]. Above all, there have been no previous reports comparing the effects of the two commonly used anesthetics, desflurane and propofol, on arterial oxygenation during OLV in thoracic surgical patients.

In accordance with previous reports [21, 28], our data demonstrated a decrease in PaO_2_ during the first 30 min of OLV and gradual improvement thereafter in both groups (Fig. 2). During desflurane anesthesia, PaO_2_ decreased significantly after 15 min of OLV compared to the pre-OLV state, and then recovered gradually to pre-OLV levels at 60 min. On the other hand, arterial oxygenation was relatively well maintained during propofol anesthesia throughout the study. None of the time points during OLV indicated a lower PaO_2_ compared to pre-OLV state in the propofol group.

One explanation for our results is that more vasodilation induced by desflurane anesthesia would have attenuated any protective effect of HPV during alveolar hypoxemia, thus decreased systemic PaO_2_ in the desflurane group compared to the propofol group. This is partially supported by significantly lower arterial blood pressure despite the lesser use in remifentanil during desflurane anesthesia compared to propofol anesthesia in the present study (Table 2). Significant drop in MAP during desflurane anesthesia compared to propofol anesthesia also has been reported in previous animal study (66 vs. 103 mmHg), in which the effect of anesthetics (desflurane vs. propofol) on arterial oxygenation during OLV was evaluated [3].

Previously, an attempt to inhibit HPV by infusing the potent vasodilator sodium nitroprusside (SNP) during OLV has failed to show significant changes in pulmonary vascular resistance (PVR), shunt fraction, or arterial oxygenation [29]. However, only seven patients were used to evaluate the effect of administering SNP on HPV, by measuring related parameters only once during OLV, before the surgery commenced. Moreover, there was no time window from drug administration to measurement of the variables, or duration of drug infusion to reach the predefined goal (25% decrease in MAP), as presented by the authors. Although vasodilation induced by SNP decreased PVR (166 ± 23–131 ± 22 dynes/s/cm^5^) and PaO_2_ (285 ± 42–225 ± 47 mmHg) in that study, the decreases were not significant. Therefore, further investigations may be needed.

Decreases in PaO_2_ and use of lung recruitment maneuvers during OLV were observed in three patients that received desflurane, while no cases were observed in the propofol group. During the events, the lowest PaO_2_ levels under F_I_O_2_ of 1.0 were 60, 69, and 87 mmHg, respectively. All episodes occurred after 30 min following initiation of OLV. In the last case, the degree of hypoxemia was not severe; however, the attending anesthesiologist decided to provide rescue treatment and proceed with OLV for the remainder of the surgical procedure. Similar events have been reported in previous animal studies comparing desflurane and propofol during in vivo OLV [3]. In this previous study, 3 of 10 pigs showed oxygen desaturation < 90% during OLV with desflurane anesthesia, while no cases were observed in the group receiving propofol. Although there were no further complications after desaturation in our study, these results suggest that desflurane anesthesia for OLV could result in deterioration of oxygenation especially in subjects with under-reserved pulmonary function.

In the current study, blood pressure was managed using adjustment of remifentanil infusion according to the hemodynamic changes during the surgical procedure. We tried to use anesthetic techniques relevant to real clinical practice. It is clearly reflected in the lower use of remifentanil in the desflurane group mainly due to the sustained lower blood pressure in this group compared to the propofol group. In a previous study investigated the effects of different concentrations of remifentanil during OLV, no differences in PaO_2_ were observed despite the significantly different blood pressures [30]. Therefore, we considered that the observed difference in PaO_2_ may not the consequence of the difference in remifentanil use between the two groups in this study.

Although a significant difference in PaO_2_ was detected between the groups (170 vs. 202 mmHg at 30 min of OLV in the desflurane vs. propofol group, respectively; *p* = 0.039), the difference may be clinically irrelevant to the anesthesiologist who is managing oxygenation in patients undergoing OLV. In this trial, we used a F_I_O_2_ of 1.0 throughout the OLV period with the expectation that it would better distinguish the difference in oxygenation between the groups [31]. A clinically significant difference in PaO_2_ may have been found between the two groups if a lower F_I_O_2_ had been used.

In some studies, desflurane showed relatively conserved vascular resistance compared to sevoflurane [32]. In other studies, desflurane has shown more vasodilatory effect among inhalational anesthetics [11–14]. Although no differences were observed in oxygenation between sevoflurane and propofol anesthesia during OLV in several previous studies [8–10], desflurane, even as balanced anesthesia, showed more impaired oxygenation during OLV compared with propofol anesthesia in the present study. However, we did not compare the two popular inhales, sevoflurane vs. desflurane, in the present trial. Further studies are required for comparison of effects of two commonly used volatile anesthetics.

The following limitations should be considered when interpreting our results. First, we did not measure the pulmonary shunt fraction, perfusion or the extent of pulmonary vasoconstriction in each isolated lung during surgery, which could have explained the effects of the anesthetic on pulmonary circulation, arteriovenous shunt fraction, or the degree of ventilation/perfusion mismatch. Therefore, it is difficult to conclude that there were any effects of the anesthetics on the role of HPV during OLV from these results. Second, we did not fix the dose of desflurane, propofol, or remifentanil during this study. Instead, they were titrated by attending anesthesiologists in charge according to anesthetic depth (BIS) or hemodynamic values. Therefore, our results do not reflect the effect of any specific anesthetic dose. Third, although we fixed F_I_O_2_ at 1.0 during OLV in both groups, minimal loss of inspired oxygen in the desflurane group was inevitable due to the volume of inhaled anesthetic. However, the present anesthetic protocol was based on the general practice of the institution. Thus, these results should be interpreted in the context of a thoracic surgery anesthesia practice using inhalational and/or intravenous anesthetics. The F_I_O_2_ or concentration of inhalational anesthetics could be adjusted according to the clinical setting. Fourth, the cardiac index used in this study was calculated based on non-externally calibrated pulse contour analysis, which may be less precise than pulmonary or transpulmonary thermodilution methods. Therefore, this may have obscured the interpretation of the effects of cardiac output on HPV. Finally, the dose of desflurane administered during the study was 1.0–1.1 MAC. This dosage may seem rather high for a balanced anesthesia protocol, reflecting the lower MAP and less use of remifentanil in the desflurane group compared to those in the propofol group. However, in real clinical situation, anesthesiologist continuously changes the dose of anesthetics according to the patient’s responses, and we believe that the results of our study may be more useful in the clinical practice.

## Conclusions

In conclusion, oxygenation under desflurane-remifentanil balanced anesthesia was lower than that under propofol-remifentanil TIVA during OLV for video-assisted thoracoscopic surgery. In patients who develop hypoxemia during OLV with inhalational anesthetics, change anesthetics to an intravenous agent should be considered.

## Abbreviations

BIS: Bispectral index
Ce: Effect site concentration
FEV_1_: Forced expiratory volume in 1 s
F_I_O_2_: Inspired oxygen fraction
FVC: Forced vital capacity
HPV: Hypoxic pulmonary vasoconstriction
MAP: Mean arterial pressure
OLV: One-lung ventilation
PaO_2_: Arterial partial pressure of oxygen
PBW: Predicted body weight
PVR: Pulmonary vascular resistance
SNP: Sodium nitroprusside
SpO_2_: Pulse oximetry
TIVA: Total intravenous anesthesia
TLV: Two-lung ventilation
